# Affordable Care Act and Disparities in Health Services Utilization among Ethnic Minority Breast Cancer Survivors: Evidence from Longitudinal Medical Expenditure Panel Surveys 2008–2015

**DOI:** 10.3390/ijerph15091860

**Published:** 2018-08-28

**Authors:** Shelley I. White-Means, Ahmad Reshad Osmani

**Affiliations:** 1 Department of Clinical Pharmacy and Translational Science, University of Tennessee Health Science Center, Memphis, TN 38163, USA; 2 Department of Economics, The University of Memphis, Memphis, TN 38152, USA; arosmani@memphis.edu

**Keywords:** disparities, breast cancer, box-cox transformation, flexible hurdle model, health care system influence

## Abstract

Breast cancer is the most prevalent female cancer in the US. Incidence rates are similar for white and black women but mortality rates are higher for black women. This study draws on rich, nationally representative data, the 2008–2015 Medical Expenditure Panel Surveys, to estimate effects of the Affordable Care Act (ACA) on reducing disparities in and access to use of diagnostic and medical services for black and Hispanic breast cancer survivors. Random effects multinomial logit, flexible hurdle and Box-Cox estimation techniques are used. The robust estimates indicate that the ACA narrowed the racial/ethnic disparity in health insurance coverage, health care utilization and out-of-pocket prescription drug expenditures among breast cancer survivors. Gaps in uninsurance significantly declined for black and Hispanic survivors. Hispanic women generally and black breast cancer survivors specifically increased use of mammography services post-ACA. The ACA did not significantly impact disparities in physician utilization or out-of-pocket prescription drug expenditures for Hispanic survivors, while there were substantive improvements for black breast cancer survivors. The paper concludes with a discussion of the strengths and limitations of the ACA for reducing disparities and improving health outcomes for a growing population of breast cancer survivors in the US.

## 1. Introduction

Breast cancer is the leading type of cancer among women of all racial and ethnic groups in the US. Although the most recent data confirm the converging incidence of breast cancer between non-Hispanic blacks (NHB) and non-Hispanic whites (NHW) [1], there are substantial disparities in breast cancer mortality between NHB and NHW. The breast cancer mortality differential between NHB and NHW women has persisted for decades. In 2012, for instance, mortality rates were 42% higher for NHB women [2]. While Hispanic women have a lower mortality and incidence rate for breast cancer than NHW and NHB women. Nonetheless, current trends in incidence rates for Hispanic women suggest concerns about future ethnic inequities in breast health outcomes. This is because from 2005 to 2014 incidence rates for Hispanic women have grown 0.3 percent each year and those of NHW women have remained stable [2].

Biological, environmental and health system factors are possible sources of these disparities. Specifically, differences in breast cancer incidence, tumor type and pathological stage at diagnosis, access to care and use of diagnostic services, delayed health care seeking behavior post abnormal mammography and sedentary lifestyles are amongst the key determinants that significantly widen the mortality gap [3]. Considering biology, NHB women are most likely to experience triple negative breast cancer, which is more aggressive and has poorer outcomes due to the lack of targeted therapies for these tumors. When screening and treatment services are used matters; mortality rates increase with stage of diagnosis. NHB women are both more likely to present for treatment at later stages of disease and have the lowest survival at each stage of diagnosis [3]. Delaying treatment for triple negative breast cancer or forgoing chemotherapy increases mortality risk. Poverty and lack of health insurance are correlated with ethnic minority status and also with limited access to breast cancer care, as well as receipt of care in resource poor settings where targeted therapies are limited [4,5]. Minority women are less likely to be serviced in facilities with digital mammography or to have breast imaging specialists reading their films [6]. Improvements in the use of mammograms and availability of radical and palliative treatments are associated with better clinical outcomes for breast cancer [7].

Reporting trends in disparities in breast cancer incidence and mortality rates are vital for understanding the challenges faced in addressing the achievement of health equity [8]. This then provides a platform for developing interventions. Since biological factors play a key role in explaining health equity differences, it is tempting to primarily focus on interventions related to specific, specialized medical treatments received by patients, such as precision medicine and clinical trial participation, as solutions [4]. But as it relates to our understanding of the causes of disparities in breast cancer outcomes (i.e., poverty, differences in access to care, differences in quality of care), health care system and health care access interventions also are needed. Health care system interventions include, health insurance coverage, health literacy, patient-clinician relationship, medical decision-making, availability of health services and quality of health care policies [9]. These are community-focused, health care environment interventions. Communities vary in how their health care systems lead to disparities and thus need diverse measures targeted to improve health equity [10].

A growing body of literature [11,12] justifies the role of using health system reforms in providing and sustaining available and affordable health care services to the entire population but most importantly to racial and ethnic minorities in the US [13]. In this paper, we examine the role of the Patient Protection and Affordable Care Act (ACA), as a strategy to improve health equity in breast health outcomes. Awareness of the ACA’s impact on the utilization of health care services by racial and ethnic minority breast cancer survivors is crucial in formulating future policy directions that aim to lower racial and ethnic mortality differentials. Nevertheless, the current literature is deficient about the possible association between the ACA and its impact on improving racial and ethnic disparities in clinical outcomes for breast cancer, a chronic health condition that requires long-term care and abundant financial resources.

Benefiting from the longitudinal dimension of Medical Expenditure Panel Survey (MEPS), a nationally representative dataset and exploiting novel estimation techniques, this study aims to explore the potential impact of health insurance reform, namely the ACA, on equitable health care services access and utilization for breast cancer survivors. Our robust estimates provide rich insight on how the ACA influenced health insurance status, use of mammograms, physician visits and out-of-pocket prescription drug expenditures among breast cancer patients. This study contributes to the literature in at least three different ways. First, to our knowledge, this is one of the first efforts to empirically analyze the possible effect of the ACA on health outcomes of NHB and Hispanic breast cancer survivors using a sizeable and representative sample size. Second, our results employ advanced statistical techniques to produce robust estimates. We implement random effects multinomial logit, Box-Cox and flexible hurdle models to estimate the effects of ACA on health insurance status, out-of-pocket prescription drug expenditures and number of doctor visits for breast cancer survivors, respectively. Finally, our analysis is built upon nationally representative datasets that clearly include pre- and post-ACA implementation periods (2008–2015). Implementation of the ACA after 2010 provides us with an exogenous policy shock during this time period. Our data include information from time periods before this shock and after this shock. Therefore, we can analyze the effect of the shock on certain health and heath care outcomes.

This paper proceeds as follows. The next sub-sections discuss the institutional setting and background information about the ACA and its provisions on breast cancer. Section 2 summarizes the data and employed estimation methods. Section 3 presents the results of the empirical models. The last two sections discuss the findings and implications of our results.

### 1.1. Provisions of the ACA and Breast Cancer Patients

The ACA is a national healthcare reform signed into law in 2010. The objective of the ACA is to increase accessibility, affordability and quality of healthcare services to millions of low- and middle-income Americans [14]. The ACA expands health insurance coverage in at least five different channels. First, it allows young adults who are under age 26 to remain covered through their parents’ plan. Second, based on income-to-poverty ratio (IPR), the ACA expands state-level Medicaid eligibility for families with IPRs of 138% or less. IPRs equal family adjusted income divided by the federal poverty threshold. In 2014, a family of four people had a federal poverty threshold of $24,008. However, this expansion entirely depends on state decision, which were made optional for states by the Supreme Court in 2012 and 2013. Third, the ACA requires every US citizen is enrolled in and maintains health insurance coverage or otherwise face a tax penalty. The penalty associated with the mandate has been set to $0 as of 2019. Fourth, it increases the number of private health insurance enrollees through provision of subsidies and tax credits. Individuals with an income level between 100% and 400% of poverty are subsidized when they obtain coverage through health insurance market places. Finally, the ACA expands coverage through an employer mandate. Middle and large size firms with at least 50 full-time employees are obligated to provide full health insurance coverage to their staff members and associated dependents (<26 years old) or otherwise pay a penalty. By 2016, only 32 states had expanded Medicaid under the ACA and the remaining states are considered “non-expansion” states.

The ACA includes provisions for wellness and prevention due to the importance of adherence to recommended screening strategies in lowering mortality for all types of diseases, especially cancers. Therefore, the ACA recommended $15 billion funding over a decade for prevention and public health missions. Also, the ACA requires all insurance plans to cover “A” and “B” rated preventive services with zero copays or deductibles [15]. “A” and “B” rated services are recommended by the US Preventive Services Taskforce as having a substantial or moderate net benefit, as confirmed by evidence-based medicine. Through income-related premium assistance and provision of financial protection, the ACA improves cost-sharing mechanisms and lowers out-of-pocket health expenditures [16]. It included several provisions aimed at meaningfully enhancing the outcomes for individuals with breast cancer, increasing overall breast health and addressing breast cancer associated disparities. The ACA addressed breast cancer challenges in at least three different interrelated areas. Access to care, prevention strategies and continuity of care, with each of these areas closely linked to breast cancer mortality disparities [17].

#### 1.1.1. The ACA and Access to Breast Care

Regarding access to care, the following provisions were included under the ACA: (1) insurance companies were not be able to deny an individual’s health coverage because he or she had breast cancer; (2) individuals with breast cancer who had been uninsured for over six months could receive coverage through high-risk pools that were established in every state; (3) Medicaid expansion options included insurance for adults under 65 who had incomes of less than 133% of the federal poverty guidelines and (4) adults who earned less than 400% of the federal poverty guidelines were allowed to qualify for discounted health insurance through government regulated insurance exchanges.

#### 1.1.2. The ACA and Preventive Breast Health

ACA strategies related to promoting breast health preventive activities included: (1) all new health insurance plans, Medicare and insurance plans offered through health insurance exchanges were required to cover preventive services recommended by the US Preventive Services Task Force, which includes mammography screening; (2) all women 40 and over with commercial health insurance plans were not required to pay an annual deductible for mammograms and Medicare recipients were not required to pay any part of the cost of their mammograms; and (3) breast feeding, which has been shown to decrease a mother’s risk for breast cancer, was supported by all non-grandfathered plans. Plans were required to provide breast feeding supplies and counseling.

#### 1.1.3. The ACA and Continuity of Care for Breast Patients

Finally, provisions associated with continuity of care are; (1) insurance companies were not able to put an annual or lifetime limit on coverage for patients with breast cancer; (2) insurance companies were not able to drop an individual from a health insurance plan because he or she had breast cancer; and (3) Medicare beneficiaries received discounts on brand-name drugs. Additionally, the prescription coverage gap created by the Medicare Part D “doughnut hole” is planned for complete closure by 2020.

Based on these changes implemented via the ACA, we test the following hypothesis. Due to the implementation of the ACA, these changes will occur: (1) an increase in health insurance coverage for breast cancer survivors post-ACA; (2) a decline in the number of uninsured breast cancer survivors; (3) an increase in use of mammograms by all women, especially breast cancer survivors; (4) an increase in the use of curative services (e.g., doctor visits) among survivors and (5) a decrease in out-of-pocket prescription drug expenditures among survivors post-ACA.

## 2. Materials and Methods

### 2.1. Data Sources

The current study utilizes the Medical Expenditure Panel Survey (MEPS) 2008–2015 datasets. MEPS is an annual survey of civilian and non-institutionalized US citizens funded by the Agency for Healthcare Research and Quality (AHRQ) since 1996. The MEPS datasets provide detailed information about the users and providers of health care services through household and insurance components, respectively [18]. The household component includes rich information on the use of health services, health expenditures, insurance plans and demographics of the survey respondents. With an overlapping design and five rounds of surveys (one baseline and four follow-ups), MEPS allows pooled cross sectional and short-panel analysis.

The key variables of interest are indicator variables that show pre- and post-ACA implementation periods. We separate the post-ACA period into two sub-periods. Sub-period A covers years 2010, 2011, 2012 and 2013, while sub-period B includes years 2014 and 2015. The 2008–2009 period is considered as the pre-ACA period and compared to both sub-periods. We include two post-ACA time periods because not all of the ACA provisions related to breast health outcomes were implemented in 2010; access related provisions 2–4 and the continuity of care provision number 3 were not implemented until 2014. Additionally, given concerns that the Great Recession of 2008 and its aftermath might confound the before-after comparison, we include a comparison between the pre-ACA period and the 2014–2015 period. The main outcome variables are health insurance type (public insurance, private insurance and uninsured), use of mammograms, number of physician visits and prescription drug expenditures. A set of demographic and socio-economic variables are included as independent variables. Table 1 shows the descriptive statistics of variables.

Each of the regression models includes variables indicating region, MSA, demographic factors (age, marital status, education) and family income. The health status variable is only included in the health insurance regression model. The insurance regression also includes number of comorbidities. The mammography usage and outpatient regressions include an employment status variable. Inclusion and exclusion of regressors in the models are based upon prior research and certain statistical procedures (e.g., testing for multicollinearity, correlation coefficient and normality assumptions).

### 2.2. Statistical Analysis

Several statistical measures are considered to explore the impact of the ACA on the health insurance coverage and utilization of preventive and curative health care services for breast cancer survivors. To better understand the ACA’s role, we separate our analysis for each racial and ethnic group; namely NHW, NHB and Hispanics. Initially, to explore the longitudinal patterns of influence, we present data in descriptive form using linear graphs. Thereafter, depending on the distribution of the dependent variables in the regressions, multinomial logit, flexible hurdle and Box-Cox statistical techniques are empirically implemented to robustly estimate the effects of the ACA on health insurance coverage, use of mammography, utilization of physician services and out-of-pocket prescription drug expenditures, respectively.

To test the hypothesis associated with the occurrence of health insurance transition pre- and post ACA we apply a random effects multinomial logistic (MNL) regression. MNL models are used when the dependent variable is categorically distributed and contain multiple choices (more than two choices). Unlike standard multinomial logit models that assume independence within a choice (multiple data points for the same choice over time) and across all the alternative choices over time, akin to Alem, et al. [19], our model relaxes the assumption of independency of multiple observations within a choice. Another important identification issue is presence of individual level heterogeneity that random effects MNL has the capability to address appropriately. We estimate the following equation:

(1) $$y_{jit}=D_{t}\beta_{j}+x_{it}^{\prime }\mu_{j}+\eta_{ji}+\upsilon_{jit}$$

where, $y_{jit}$ is the type of health insurance j held by individual i at time t and $x_{it}^{\prime }$ is a vector of independent variables and $\upsilon_{jit}$ is idiosyncratic error that follows independent and identical distribution. $D_{t}$, is a binary variable that shows the pre- and post-ACA periods. Finally, $\eta_{ji}$ is individual-specific random effects that capture unobserved heterogeneity. In our model, the choice of holding private health insurance is considered as comparison group.

To test the hypothesis on the use of mammogram pre- and post-ACA, we empirically estimate random effects logistic regression. Here the dependent variable is dichotomous. The woman was asked if she had a mammogram in the last two years. If the response is yes, we coded it 1 and otherwise 0. The empirical model takes the following form:

(2) $$\ln\left(\frac{P(y_{it}=1|x_{it\;}u_{t})}{P(y_{it}=0|x_{it\;}u_{t})}\right)=\rho_{0}+D_{t}\rho_{1}+\overset{S}{\underset{s=2}{\sum }}x_{it}\rho_{s}+u_{t}+\epsilon_{it}$$

where $y_{it}$ is a dichotomous variable that indicates mammogram use or not, $x_{it}$ is a set of socio-economic covariates, $D_{t}$ is the dummy shows pre- and post ACA periods, $u_{t}$ is the random effect that is assumed to follow normal distribution and $\epsilon_{it}$ is the error term.

Next, besides changes in the use of preventive services, such as mammograms, as a result of health insurance changes, we hypothesize the possible improvement in the utilization of physician visits by breast cancer survivors. Use of physician services is reflected by count data. An excess number of zeros and the presence of strictly positive data points in the count datasets simply violate the main assumptions of standard Ordinary Least Square (OLS) specifications. One of the basic assumptions of OLS models is the normal distribution of the residual errors. For skewed continuous dependent variables, transformation of the dependent variable may produce errors that are approximately normal [20]. However, in the case of zero data points, categorical and discrete choices, transformation of dependent variables does not provide normally distributed error terms. Moreover, in such circumstances, OLS models produce negative predicted values that are theoretically incorrect [21,22]. Recent literature has seen significant improvements in modelling count data by applying hurdle models [23,24,25]. To appropriately address the unobserved heterogeneity in the utilization of curative care and to provide a flexible modelling strategy, we implement zero-truncated Poisson log-normal regression models [26]. The log-likelihood function of the above model is shown as it follows:

(3) $$LL=\overset{\infty }{\underset{-\infty }{\int }}\frac{\exp\left(-\exp\left(x_{i}\tau +\sigma \varepsilon_{i}\right)\right)exp{\left(x_{i}\tau +\sigma \varepsilon_{i}\right)}^{y_{i}}}{\left(1-\exp\left(-\exp\left(x_{i}\tau +\sigma \varepsilon_{i}\right)\right)\right)y_{i}!}\phi \left(\varepsilon_{i}\right)d\varepsilon_{i}$$

In Equation (3), τ is the parameter to be estimated. The $\phi \left(\varepsilon_{i}\right)$ is the density function of standard normal distribution. The outcome variable (OOP prescription drug expenditure) is denoted by $y_{i}$ and $\varepsilon_{i}$ is the distributed error term.

Finally, we expect changes in out-of-pocket prescription drug expenditures post implementation of the ACA among breast cancer survivors. Modelling prescription drug expenditures requires much attention be given to the skewed distribution of the data points [27]. Past literature has heavily focused on log-transformations of expenditures or two-part modelling techniques [28,29,30]. However, the weak identification assumptions in each of the above methods provide less reliable estimates [31]. We empirically estimate the effects of the ACA on out-of-pocket prescription drug expenditures by applying a left-hand side Box-Cox power transformation [32]. This method has certain strengths that surpasses OLS models. First, unlike a log-transformation, it is much more flexible in terms of choosing powers for the skewed dependent variable [33]. Second, it provides more stabilized variances for the estimated coefficients [34]. The empirical model of interest takes the following form:

(4) $$y_{i}^{\left(\lambda \right)}=\delta_{0}+\overset{P}{\underset{k=1}{\sum }}\delta_{k}x_{it}^{\prime }+\upsilon_{i}$$

(5) $$y_{i}^{\left(\lambda \right)}=\frac{y_{i}^{\left(\lambda \right)}-1}{\lambda }$$

(6) $$y_{i}^{\left(\lambda \right)}=[\begin{array}{ll} \;\;\;\;\frac{y_{i}^{\left(\lambda \right)}-1}{\lambda } & \lambda \neq 0\\ log\;y_{i} & \lambda =0\\ \;\;\;y_{i} & \lambda =1 \end{array}$$

In Equations (3) and (4), $y_{i}^{\left(\lambda \right)}$ is out-of-pocket prescription drug expenditures that are transformed with the power of λ and $\upsilon_{it}$ is the error term. We report the result of Equation (3) in the results section of this paper. Also, the applied transformation is tested against base and log-forms as well.

The key assumption of our identification strategy is based on the fact that health insurance coverage, utilization of health care services and out-of-pocket health care expenditures would have followed similar trends among all racial and ethnic groups in the absence of ACA, which is an exogenous policy shock. By inspecting the graphs of all outcome variables before and after ACA implementation, we conclude that prior to 2010, racial and ethnic differences in outcomes were far from convergence and moving with a similar trend.

## 3. Results

### 3.1. Uninsurance and the ACA

**Figure 1:**
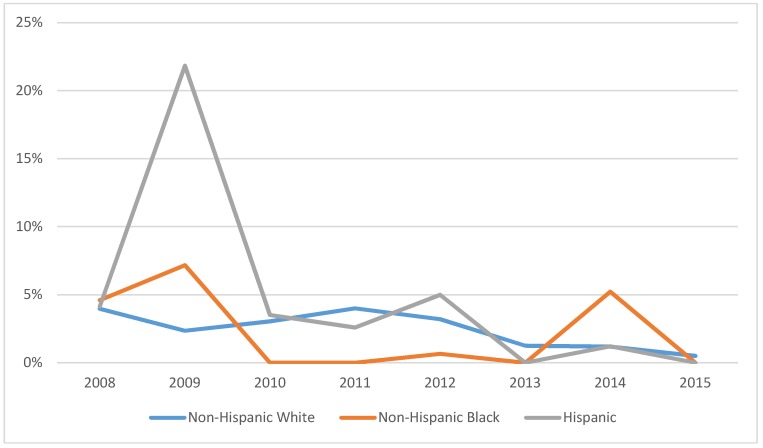
Rates of uninsured breast cancer survivors by race/ethnicity—MEPS 2008–2015 (N = 1401).

Figure 1 depicts trends in uninsured status by race/ethnicity, from 2008 to 2015. The raw data indicate a disparity in health insurance coverage, with NHW least likely to be uninsured in the pre-ACA period. In 2010, the uninsured rates for NHB and Hispanics sharply declined, with the uninsured rate for NHB approximately zero. By 2011, NHW had the highest uninsured rate. By 2015, however, the uninsured rate converged to zero for all racial and ethnic groups. To complement the preliminary findings of our raw data analysis, we empirically estimate random effects multinomial logit (MNL) model and the results are presented in Table 2 and Table 3 for the sub-periods A and B, respectively. For comparison, see Table A1 that reports public insurance coverage and uninsurance regression estimates using a logistic regression procedure that does not account for random effects. The random effects MNL model includes a dummy variable to account for the pre- or post-ACA time periods and controls for regional differences, socio-economic characteristics, health status and number of comorbidities. The table reports the relative risk ratios for a unit change in the predictor variable.

Regardless of the post ACA sub-periods, the estimated coefficients of regressions indicate that the expected risk that NHB and Hispanic breast cancer survivors are uninsured is significantly lower post-ACA. In the first sub-period, although the relative risk of being uninsured is lower for both NHB and Hispanic breast cancer survivors, the gain is higher for NHB survivors. In the second sub-period, the risk of being uninsured declined for all racial and ethnic groups, with the gains highest for Hispanic survivors. Consistent with past literature [35,36] on the coverage effect of the ACA, we reject the null hypothesis that the ACA has not improved health insurance coverage for racial and ethnic minority breast cancer survivors.

### 3.2. Mammography and the ACA

**Figure 2:**
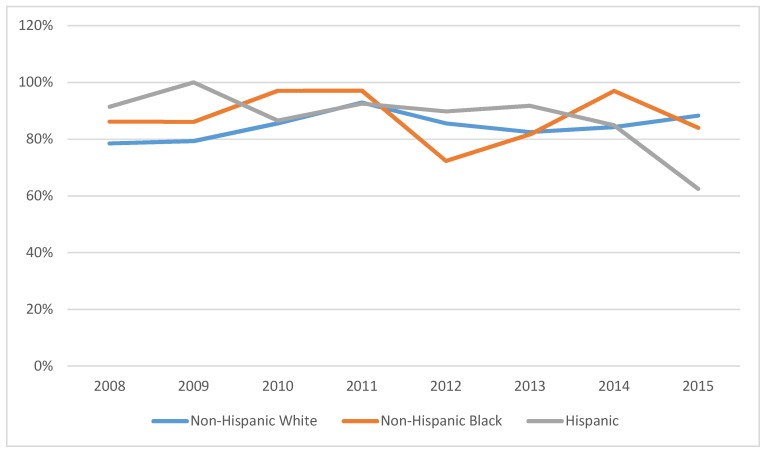
Percent breast cancer survivors having mammogram procedure in the last two years, by race/ethnicity—MEPS 2008–2015 (N = 1401).

Figure 2 illustrates trends in mammography use among breast cancer survivors by race/ethnicity, from 2008 to 2015. The raw data reveal that among breast cancer survivors, NHB and Hispanic survivors were more likely than NHW survivors to use mammograms pre-ACA. However, by 2012, NHB survivors had lower use and by 2015 Hispanic survivors had lower use than NHW survivors.

Table 4 reports the random effects logistic regression results for mammography use within the last two years for breast cancer survivors. For comparison, see Table A2 that reports mammography utilization regression estimates using a logistic regression procedure that does not account for random effects. The regression models show that post-ACA the odds of mammography use were 1.1 times higher among NHB women during post-ACA subperiod A. During subperiod B, NHW and NHB had odds of mammography use that were 1.5 and 1.3 times higher. The subperiod B results were marginally significant among Hispanic women. These data are suggesting that disparities in mammography use decreased between NHW and NHB women.

**Figure 3:**
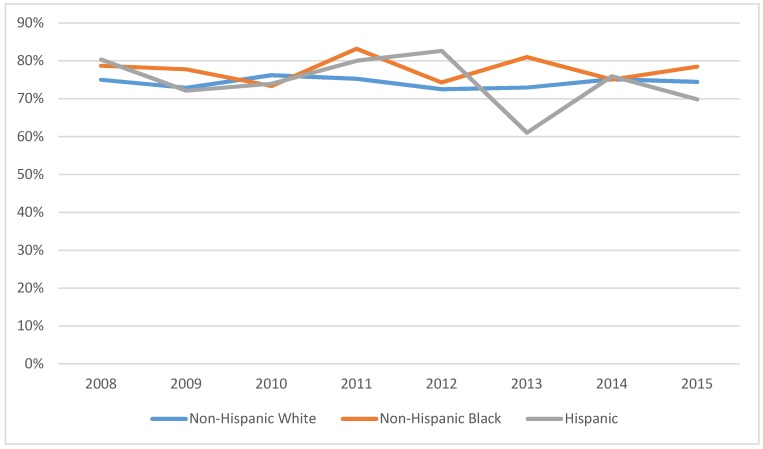
Percent of women age 21–65 having mammogram procedures in the last two years, by race/ethnicity—MEPS 2008–2015 (N = 9613).

Use of mammograms is not only crucial for breast cancer survivors but it significantly improves early detection and better clinical outcome for all women of child-bearing age. In an effort to test the possible improvement in overall utilization of mammograms as a result of the ACA, we report the trend analysis depicted in Figure 3 that explores disparities in mammography use for different racial and ethnic groups between 2008 and 2015. It seems that the ACA minimized the gap in mammography utilization among all racial and ethnic groups with an exception for Hispanic women that showed a sharp decline in use in 2013. To support the conclusions reached via the raw data trend analysis, we implement another random effects logistic regression model for the sample of women of all ages and include an interaction term between a dummy variable indicating whether the woman is a breast cancer survivor and time dummy that shows pre- and post-ACA (Table 5). This is because we suspect that mammography use may be different among women who have experienced breast cancer compared with women who are cancer free.

Comparing the post-ACA subperiod A to the pre-ACA time period, there were no differences in mammography use for any racial or ethnic group. During post-ACA time period B, Hispanic women were 7 times more likely to have a mammogram than during the pre-ACA period. The odds of mammography use were 14.6 and 17.2 times higher for Hispanic women with breast cancer when compared to cancer-free Hispanic women in the two post-ACA periods. The likelihood of using mammography by NHW and NHB breast cancer survivors were 2.1 and 2.5 times higher, respectively, than for cancer-free NHW and NHB women during post-ACA period A. Similarly, comparing the post-ACA subperiod B mammography use to that of the pre-ACA period, NHW and NHB breast cancer survivors use were 1.1 and 1.0 times higher during the later period or cancer-free NHW and NHB women during post-ACA period A. In sum, all Hispanic women, survivors and those cancer free, were much more likely to use mammograms during the post-ACA subperiod B than they were pre-ACA. For NHW and NHB women, higher use of mammography occurred during the post-ACA period for breast cancer survivors only but not for cancer free NHW and NHB women.

### 3.3. Physician Services Utilization and the ACA

**Figure 4:**
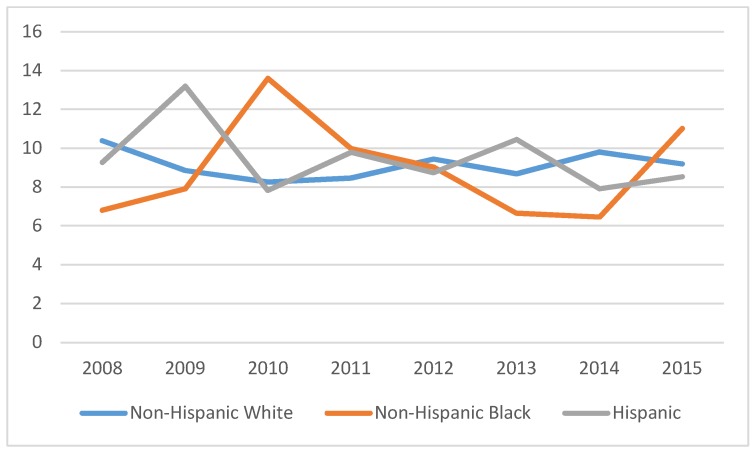
Average number of physician visits among breast cancer survivors, by race/ethnicity, MEPS 2008–2015 (N = 4947).

Figure 4 reports racial/ethnic patterns in physician visits between 2008 and 2015. Physician visits were relatively stable throughout the period for NHW women. Hispanic women tended to have greater or equivalent visits as NHW women. However, for NHB women, utilization was generally lower than that of NHW women, with the exception of the 2009–2011 periods.

Empirically speaking, the physician visit is a combination of two different decisions with separate data generating processes [37]. The first decision is to visit the physician and the second is the number of times to use physician visits. Figure 4 reports the final outcome of the two decisions. Table 5 reports the regression results that account for the two-part decision to use physician services, that is, it reports the factors influencing the number of physician visits controlling for the role of these factors in influencing the decision to use or not use physician services. The regression is a zero-truncated model that assumes the truncated data are distributed according to a Poisson distribution and the errors are log-normally distributed [26]. These estimates are robust, with marginal effects reported in Table 6. For comparison, see Table A3 that reports Ordinary Least Squares regression estimates for physician visits and does not account for the two-part decision.

According to the results reported in Table 6, there is some support for a narrowing of the racial/ethnic gap in physician services use due to the ACA. The data indicate that post-ACA, NHB and NHW breast cancer survivors were more likely to have physician visits than pre-ACA, while the effect was larger for NHB survivors. Comparing the post-ACA subperiod B with the Pre-ACA period, NHB survivors were more likely to have physician visits, with the marginal effect higher in post-ACA period B than in post-ACA period A. There were no significant differences in Hispanic breast cancer survivors’ physician visits post-ACA compared with the pre-ACA period.

### 3.4. Prescription Drug Expenditures and the ACA

**Figure 5:**
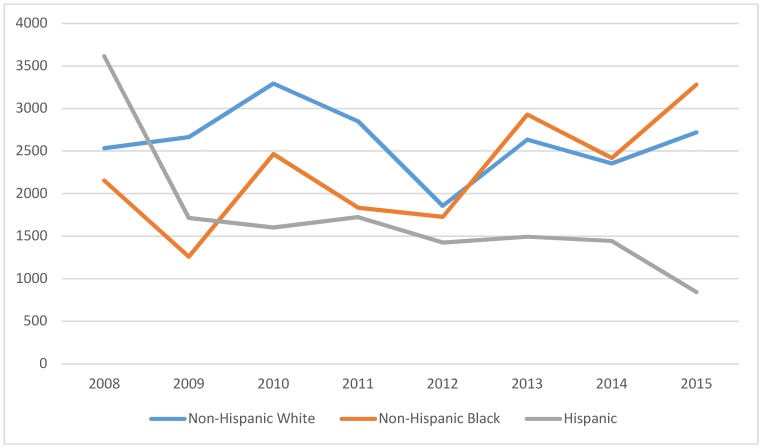
Average out-of-pocket prescription drug expenditures by breast cancer survivors across race/ethnicity—MEPS 2008–2015 (N = 836).

Another component of health care utilization is prescription drug expenditures. In MEPS, these expenditures include patient out-of-pocket payments, payments made by private insurance, Medicaid, Medicare and other sources. We report on out-of-pocket expenditures because they influence whether health care access is or is not be impeded and whether patients will have enough residual financial resources in order to finance other non-health care necessities. NHB and Hispanic cancer survivors have a higher risk of noncompliance with medication regimens when out-of-pocket medication costs are too high [38]. Figure 5 reports trends in out-of-pocket expenditures for prescription drugs and indicates that from 2010 to 2015, Hispanic breast cancer survivors had lower out-of-pocket expenditures than either NHB or NHW breast cancer survivors, with NHW out-of-pocket expenditures the highest. After 2012, out-of-pocket drug expenditures for NHB breast cancer survivors exceeded those of NHW and Hispanic breast cancer survivors.

**Figure 6:**
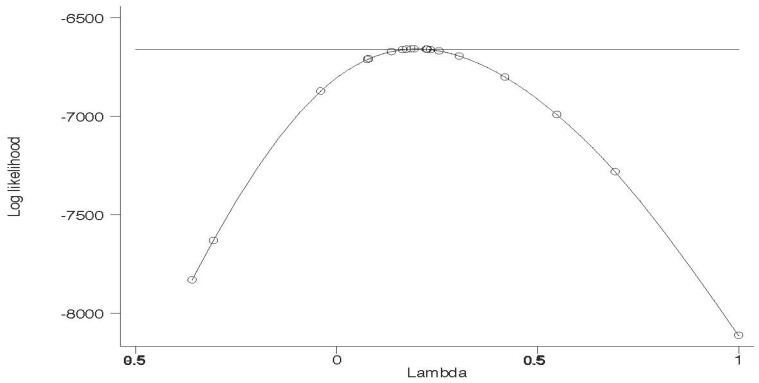
Log-Likelihood test of Lambda (power of transformation) for Non-Hispanic White.

**Figure 7:**
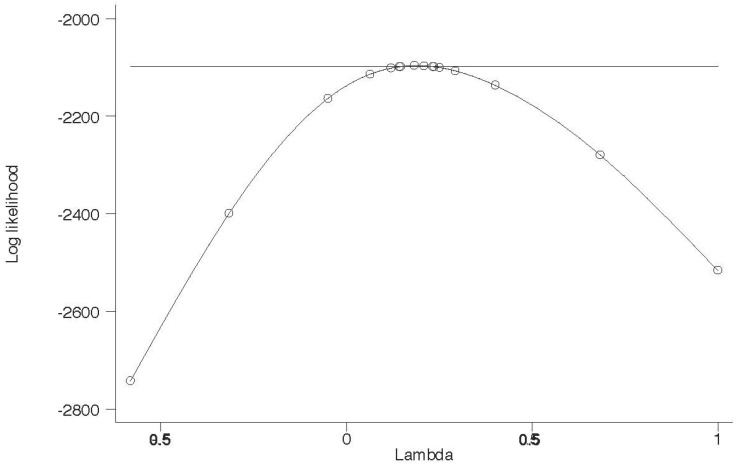
Log-Likelihood test of Lambda (power of transformation) for Non-Hispanic Black.

**Figure 8:**
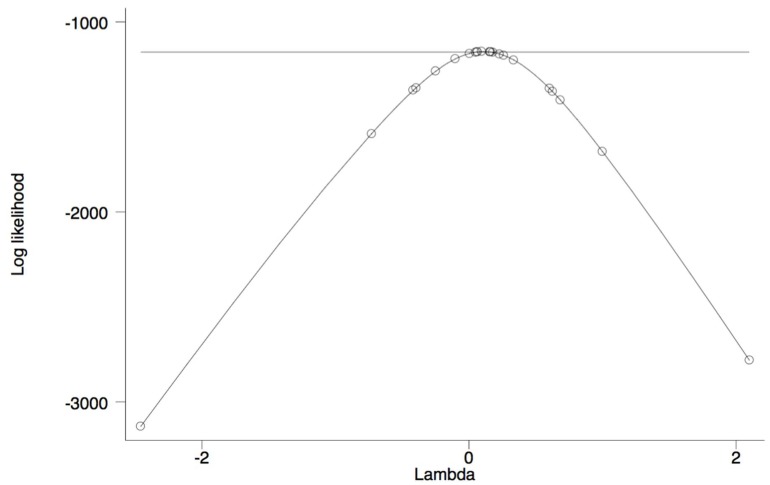
Log-Likelihood test of Lambda (power of transformation) for Hispanic.

Exploring out-of-pocket prescription drug expenditures within a regression framework generates a slightly different conclusion related to changes in disparities in out-of-pocket prescription drug expenditures. Table 7 reports the results of a Box-Cox regression of out-of-pocket prescription drug expenditures. The graph of power transformation for each model associated with respective racial and ethnic groups is shown by Figure 6, Figure 7 and Figure 8. The Box-Cox powers are plotted against the value of the log-likelihood. As shown, the monotonic non-linear transformation is not rejected in favor of the base form and semi- log-transformation at 0.01% level of significance. For comparison, see Table A4 that reports Ordinary Least Squares regression estimates for out-of-pocket prescription drug expenditures and does not use the Box-Cox procedure.

Post ACA, out-of-pocket prescription drug expenditures for NHB breast cancer survivors decreased significantly, thus narrowing racial disparities in out-of-pocket prescription drug expenditures.

## 4. Discussion

This paper explores the extent that the ACA, a system level intervention that focused on prevention and access to and continuity of care, could improve racial and ethnic equity in health care for breast cancer survivors. The robust estimates produced in these analyses using the longitudinal dimensions of MEPS 2008–2015 indicate that the ACA narrowed the racial and ethnic disparity in health insurance coverage, health care utilization and out-of-pocket prescription drug expenditures among the breast cancer survivors.

Our findings support hypotheses 1 and 2. The ACA led to an increase in insurance and a decline in uninsurance during both sub-period A and sub-period B for NHB and Hispanic breast cancer survivors. NHW survivors experienced declines in uninsurance in sub-period B only. The support for Hypothesis 3 was contingent on the post-ACA time period considered and varied by race/ethnicity. The hypothesis was supported for all Hispanic women, survivors and those cancer free, during the post-ACA subperiod B; they were much more likely to use mammograms than they were pre-ACA. Mammogram use for all NHW and NHB women did not increase post-ACA. Only NHB and NHW survivors had higher use of mammography and this occurred during both post-ACA periods. Hypothesis 4 was confirmed for NHB and NHW breast cancer survivors during post-ACA period A and for NHB survivors during post-ACA period B. However, there were no significant differences in Hispanic breast cancer survivors’ physician visits post-ACA compared with the pre-ACA period. Hypothesis 5, a decrease in out-of-pocket prescription drug expenditures among survivors post-ACA was supported by or findings but only for NHB survivors.

The ACA impacted health care equity for NHB and Hispanic breast cancer survivors differently. The ACA improved equity in insurance coverage but had larger effects on NHB survivors compared to Hispanic breast cancer survivors during post-ACA subperiod A, while during post-ACA subperiod B the effects were larger for Hispanic survivors. Hispanic women generally increased mammography use post-ACA, while only NHB breast cancer survivors increased mammography use post-ACA. The ACA did not increase physician utilization for Hispanic breast cancer survivors, yet utilization increased for NHB survivors. The ACA significantly reduced out-of-pocket drug expenditures for NHB survivors but only marginally decreased out-of-pocket drug expenditures for Hispanic survivors.

Disaggregating the data in ways that we could explore the impact of the ACA in the early years versus the later periods was also important. The ACA implemented changes in access to preventive services and coverage for uninsurables during the early periods. In the later period (2014–2015), the ACA implemented Medicaid expansion, changes in the price of insurance coverage for those with incomes less than 400% of poverty and Medicare discounts. The early ACA period was associated with larger changes in the uninsured, primarily for NHB and Hispanic survivors. The later period reduced uninsurance for all groups. ACA changes made during the later time period significantly increased Hispanic survivors’ use of mammograms. ACA changes made during both time periods were important for increasing NHB survivors’ physician visits.

It is important to explore further the reason that only marginally significant decreases in disparities in out-of-pocket drug utilization and no changes in physician visits occurred post-ACA for Hispanic breast cancer survivors. Maybe in this case, other barriers to utilization, such as challenges in obtaining transportation to facilities and racial/ethnic differences in breast cancer treatment strategies, have some influences on this outcome.

A strength of our findings is that they are not solely based on self-reports. MEPS verifies insurance coverage, medical visits and all medical expenditures with provider claims data and other formal records. In addition, by using advanced statistical techniques, our estimates are robust.

One limitation of our analysis is that we do not have community level data that would allow us to discern the roles of factors, such as travel distance to facilities, in influencing decisions about physician services and mammography use. Also unknown are data on characteristics of physicians, presence of language barriers and types of social support available to the women as they seek to improve their health by utilizing the privileges provided by the ACA.

## 5. Conclusions

These results add to a growing body of literature that indicates that the ACA has had a positive impact on health care access and health outcomes [7,13,17,36,39]. We note that the ACA has also impacted another social concern for our health care system, racial and ethnic health care equity. Challenges to maintaining ACA health care equity gains exist. Two of these are proposals to remove the health insurance coverage mandate and the requirement that preexisting conditions do not exclude one from health insurance coverage. These provisions allowed the ACA to maintain affordable premiums and expand health insurance coverage to all income groups. Documenting the health equity impact of the various provisions of the ACA not only informs about its derived benefits but also, we discern the potential costs/negative repercussions of the legislations’ repeal or modifications to specific provisions.

## Figures and Tables

**Table 1:** Descriptive summary and characteristics of breast cancer survivors—MEPS 2008–2015.

	Pre-ACA			Post-ACA		
	Non-Hispanic White (%)	Non-Hispanic Black (%)	Hispanics (%)	Non-Hispanic White (%)	Non-Hispanic Black (%)	Hispanics (%)
Survivor’s age	66.45 (12.87) ^a^	60.15 (13.04)	57.64 (15.16)	67.15686 (12.38468)	62.673 (12.74)	59.64 (14.30)
Doctor’s visit	9.55 (9.88)	6.80 (7.93)	10.6 (13.98)			
OOP prescription Expenditure	2936 (3303)	2110 (2893)	3620 (6320)	2902.5 (6725)	2554 (4790)	3999 (19,678)
Number of priority conditions	1.82 (1.54)	1.77 (1.43)	1.64 (1.67)	1.78 (1.482)	2.33 (1.58)	1.51 (1.150)
Mammography within 2 years						
No	20.88	15.00	7.14	14.67	12.08	14.05
Yes	79.12	85.00	92.86	85.33	87.92	85.95
Health insurance						
Private insurance	63.60	47.13	33.33	65.61	54.84	45.74
Public insurance	32.51	45.98	53.33	31.37	43.78	48.84
Uninsured	3.89	6.90	13.33	3.02	1.38	5.43
Census regions						
Northeast	18.73	18.39	20.00	16.44	14.29	24.81
Midwest	24.03	17.24	4.44	27.00	15.21	7.75
South	35.69	52.87	35.56	36.20	60.37	31.78
West	21.55	11.49	40.00	20.36	10.14	35.66
Marital status						
Married	56.54	29.89	42.22	52.34	29.95	48.84
Widowed/Divorced	39.58	49.43	40.00	41.48	55.76	43.41
Never married	3.89	20.69	17.78	6.18	14.29	7.75
Education						
HS and GED	66.78	71.26	80.00	50.23	60.83	67.44
Bachelor	16.96	16.09	15.56	35.75	29.49	27.13
Graduate	16.25	12.64	4.44	14.03	9.68	5.43
Health status						
Excellent/very good	42.65	26.74	22.22	47.09	29.30	26.98
Good/fair	50.90	63.95	68.89	46.32	57.67	64.29
Poor	6.45	9.30	8.89	6.60	13.02	8.73
Family income						
Low income	25.80	45.98	44.44	32.43	45.16	53.49
Middle income	31.10	26.44	44.44	26.40	32.72	24.81
High income	43.11	27.59	11.11	41.18	22.12	21.71
Employment status						
Unemployed	62.41	54.02	66.67	65.20	63.89	58.14
Employed	37.59	45.98	33.33	34.80	36.11	41.86
MSA	80.21	22.06	100.00	79.59	90.08	96.43
Non-MSA	19.79	15.15	0.0	20.41	9.92	3.57
Number of observations	283	87	45	663	217	129

Metropolitan Statistical Area (MSA), General Educational Development (GED), High School (HS). ^a^ Standard error are shown in the parenthesis for the continuous variables. Mean of continuous variables is reported.

**Table 2:** Random effects multinomial logistic regression analysis of public insurance coverage and uninsurance, by race/ethnicity—sub-sample A (2008–2013).

	Non-Hispanic White		Non-Hispanic Black		Hispanic	
	Public Insurance	Uninsured	Public Insurance	Uninsured	Public Insurance	Uninsured
Variables	(1)	(2)	(3)	(4)	(5)	(6)
Sub period A ^a^	0.881	1.329	0.509 *	0.0252 **	0.361 *	0.372 ***
	(0.168)	(0.628)	(0.194)	(0.0374)	(0.199)	(0.150)
Survivor’s age	1.522 ***	0.496 ***	1.425 **	1.034	1.452 *	0.439 **
	(0.138)	(0.0822)	(0.241)	(0.351)	(0.322)	(0.166)
Census regions (Northeast †)						
Midwest	1.064	4.847	2.303	4.72 × 10^−7^ ***	0.163	0.404
	(0.300)	(5.731)	(1.413)	(7.15 × 10^−7^)	(0.245)	(0.584)
South	1.003	6.386	1.248	2.317	0.775	1.904
	(0.262)	(7.361)	(0.661)	(2.810)	(0.536)	(1.941)
West	0.948	5.164	0.305	1.54 × 10^−7^ ***	0.175 **	0.895
	(0.286)	(6.117)	(0.231)	(1.82 × 10^−7^)	(0.138)	(0.750)
Marital status (Married †)						
Widow/divorced	1.582 **	1.161	2.158 *	0.279	5.620 ***	1.509
	(0.314)	(0.575)	(0.906)	(0.360)	(3.347)	(1.393)
Never married	2.856 ***	8.374 ***	2.173	0.163	1.501	0.364
	(1.124)	(6.480)	(1.127)	(0.208)	(1.025)	(0.460)
Education (HS & GED †)						
Bachelor	0.967	0.256 **	0.355 **	1.25 × 10^−7^ ***	0.746	1.030
	(0.218)	(0.159)	(0.166)	(1.43 × 10^−7^)	(0.499)	(0.951)
Graduate	0.711	0.453	0.933	3.79 × 10^−7^ ***	0.0682	0.908
	(0.224)	(0.329)	(0.523)	(5.36 × 10^−7^)	(0.120)	(1.329)
Health status (Excellent/very good †)						
Good/fair	0.902	1.160	1.505	0.444	1.205	0.322
	(0.181)	(0.637)	(0.672)	(0.527)	(0.810)	(0.277)
Poor	1.368	9.90 × 10^−7^ ***	4.948 **	3.286	3.901	1.299
	(0.511)	(8.73 × 10^−7^)	(3.382)	(7.547)	(6.689)	(2.062)
Number of priority conditions	1.175 **	0.817	1.371 **	1.029	1.029	0.969
	(0.0814)	(0.167)	(0.190)	(0.523)	(0.244)	(0.335)
Family income as % FP line (Low income †)						
Middle income	0.447 ***	2.803 *	0.218 ***	0.183 *	0.116 ***	1.064
	(0.1000)	(1.614)	(0.0841)	(0.175)	(0.0680)	(0.840)
High income	0.264 ***	0.717	0.0471 ***	3.53 × 10^−8^ ***	0.132 ***	0.214
	(0.0620)	(0.474)	(0.0265)	(4.52 × 10^−8^)	(0.0966)	(0.212)
Constant	0.0379 ***	0.713	0.0911 **	2.732	0.722	67.20 *
	(0.0256)	(1.096)	(0.110)	(8.586)	(1.082)	(149.1)
Observations	699	699	241	241	123	123
*Wald* $\chi^{2}$ *Test*	623 ***	623 ***	342 ***	342 ***	199 ***	199 ***
σ	5.45	5.45	3.75	3.75	2.03	2.03
	(2.13)	(2.13)	(1.19)	(1.19)	(0.45)	(0.45)
ρ	0.854	0.854	0.572	0.572	0.113	0.113
*Wald* $\chi^{2}$ *Test*	791 ***	791 ***	515 ***	515 ***	211 ***	211 ***

Relative Risk ratio and linearized standard error are reported. Standard errors are reported in parenthesis and clustered at the individual level. High School (HS), General Education Diploma (GED). * *p* < 0.10, ** *p* < 0.05, *** *p* < 0.01; † Shows the base (comparison) category; ^a^ Equals 1 if years are 2010, 2011, 2012, 2013 and 0 if years are 2008 and 2009.

**Table 3:** Random effects multinomial logistic regression analysis of public insurance coverage and uninsurance, by race/ethnicity—sub-period B.

	Non-Hispanic White		Non-Hispanic Black		Hispanic	
	Public Insurance	Uninsured	Public Insurance	Uninsured	Public Insurance	Uninsured
Variables	(1)	(2)	(3)	(4)	(5)	(6)
Sub period B ^a^	0.837	0.633 ***	0.270 ***	0.315 *	0.259 **	0.0496 **
	(0.215)	(0.0840)	(0.0829)	(0.210)	(0.169)	(0.0671)
Survivor’s age	1.668 ***	0.667 **	2.454 ***	0.680	3.036 ***	0.468
	(0.180)	(0.132)	(0.777)	(0.309)	(1.223)	(0.255)
Census regions (Northeast †)						
Midwest	1.024	2.660 × 10^6^ ***	0.401	2.75 × 10^−8^ ***	0.000169 ***	7.257
	(0.346)	(1.755 × 10^6^)	(0.418)	(4.35 × 10^−8^)	(0.000337)	(18.10)
South	0.635	3.036 × 10^6^ ***	0.170 *	0.402	0.0571 **	0.404
	(0.200)	(2.115 × 10^6^)	(0.169)	(0.514)	(0.0639)	(0.819)
West	0.996	3.944 × 10^6^ ***	0.0590 **	1.46 × 10^−8^ ***	0.00863 ***	0.0348 **
	(0.345)	(3.069 × 10^6^)	(0.0731)	(1.81 × 10^−8^)	(0.00954)	(0.0516)
Marital status (Married †)						
Widow/divorced	1.715 **	1.461	1.672	0.868	17.53 ***	3.795
	(0.408)	(1.007)	(0.979)	(1.467)	(16.42)	(4.128)
Never married	2.039	8.587 **	10.36 ***	0.851	17.33	6.008
	(1.138)	(8.374)	(7.517)	(1.291)	(31.70)	(10.23)
Education (HS & GED †)						
Bachelor	0.534 **	0.562	0.194 ***	2.07 × 10^−8^ ***	3.413	1.429
	(0.157)	(0.414)	(0.107)	(1.66 × 10^−8^)	(4.151)	(2.661)
Graduate	0.484 *	0.864	0.631	5.21 × 10^−8^ ***	0 ***	2.917
	(0.180)	(0.800)	(0.519)	(7.47 × 10^−8^)	(0)	(6.339)
Health status (Excellent/very good †)						
Good/fair	1.158	1.275	2.089	0.401	110.8 ***	0.733
	(0.285)	(0.855)	(1.409)	(0.520)	(145.9)	(0.907)
Poor	1.104	2.149	2.647	2.49 × 10^−8^ ***	2.270 × 10^8^ ***	1.439 × 10^9^ ***
	(0.537)	(2.540)	(2.768)	(4.62 × 10^−8^)	(2.823 × 10^8^)	(2.863 × 10^9^)
Number of priority conditions	1.142 *	1.076	1.278	1.118	0.601	0.182
	(0.0922)	(0.255)	(0.191)	(0.366)	(0.212)	(0.234)
Family income as % FP line (Low income †)						
Middle income	0.325 ***	2.905	0.163 ***	0.481	0.0265 ***	6.017
	(0.0968)	(2.595)	(0.106)	(0.715)	(0.0273)	(8.495)
High income	0.237 ***	0.534	0.0552 ***	1.14 × 10^−8^ ***	0.0340 **	3.65 × 10^−6^ ***
	(0.0691)	(0.564)	(0.0447)	(1.60 × 10^−8^)	(0.0492)	(5.48 × 10^−6^)
Constant	0.0291 ***	1.17 × 10^−7^ ***	0.0171 **	42.37	0.00975 **	30.62
	(0.0235)	(1.91 × 10^−7^)	(0.0332)	(139.9)	(0.0223)	(78.58)
Observations	507	507	145	145	93	93
Wald $\chi^{2}$ Test	421 ***	421 ***	273 ***	273 ***	201 ***	201 ***
Σ	3.77	3.77	2.98	2.98	3.82	3.82
	(2.01)	(2.01)	(0.98)	(0.98)	(0.33)	(0.33)
ρ	0.616	0.616	0.411	0.411	0.102	0.102
Wald $\chi^{2}$ Test	631 ***	631 ***	411 ***	411 ***	111 ***	111 ***

Relative Risk ratio and linearized standard errors are reported. Standard errors are reported in parenthesis and clustered at the individual level. High School (HS), General Education Diploma (GED). * *p* < 0.10, ** *p* < 0.05, *** *p* < 0.01; † Shows the base (comparison) category; ^a^ Equals 1 if years are 2014, 2015 and 0 if years are 2008 and 2009.

**Table 4:** Random effects logistic regression of mammography use among breast cancer survivors, by race/ethnicity—MEPS 2008–2015.

	Pre-ACA and Sub-Period A Sample			Pre-ACA and Sub-Period B Sample		
	(Non-Hispanic White)	(Non-Hispanic Black)	(Hispanic)	(Non-Hispanic White)	(Non-Hispanic Black)	Hispanic
Variables	Mammography (1)	Mammography (2)	Mammography (3)	Mammography (4)	Mammography (5)	Mammography (6)
ACA periods (Time dummies)						
Sub period A ^a^	3.093 *	1.107 ***	0.00219	----	---	---
	(1.882)	(0.399)	(0.0137)	---	---	---
Sub Period B ^b^	---	---	---	1.486 **	1.255 **	0.870 *
	---	---	---	(0.647)	(0.533)	(0.489)
Survivor’s age	1.059	0.054 **	3.112	1.066	1.167	1.486 **
	(0.262)	(0.025)	(4.584)	(0.356)	(1.823)	(0.647)
Census regions (Northeast †)						
Midwest	0.190 *	0.204 *	0.729 **	0.433	0.510	0.315 *
	(0.191)	(0.121)	(0.365)	(0.561)	(0.612)	(0.210)
South	0.391	0.108	2.600	0.255	1.464 *	0.669
	(0.346)	(0.240)	(10.10)	(0.343)	(0.822)	(0.463)
West	0.126 *	0.698	2.508	0.0758	0.0833	0.633 ***
	(0.137)	(1.601)	(11.42)	(0.134)	(0.712)	(0.0840)
MSA (non-MSA †)	0.851	0.847 *	2.876 **	1.046		0.229
	(0.525)	(0.528)	(1.239)	(0.891)		(0.407)
Marital status (Married †)						
Widow/divorced	0.676	0.042 *	38.70	1.059	0.00325	1.021
	(0.380)	(0.023)	(174.9)	(0.793)	(0.0195)	(0.0203)
Never married	0.210	0.762	0.809 *	0.132	0.00748	1.020
	(0.251)	(1.518)	(0.450)	(0.254)	(0.0446)	(0.355)
Education (HS & GED †)						
Bachelor	0.243 *	1.290	3.268 **	0.195	0.0295	0.715
	(0.182)	(1.817)	(1.512)	(0.242)	(0.136)	(0.270)
Graduate	5.863	3.159	0.406	2.782 ***	0.333 **	0.834
	(6.641)	(6.403)	(1.408)	(1.074)	(0.153)	(0.536)
Number of priority conditions	0.893	1.665	0.0707	1.205	47.76	0.715
	(0.159)	(0.844)	(0.177)	(0.325)	(167.5)	(0.270)
Family income as % FP line (Low income †)						
Middle income	0.893	1.090	0.00319	0.091 *	0.059 *	0.005
	(0.552)	(1.399)	(0.0196)	(0.058)	(0.032)	(0.081)
High income	0.941	0.059 *	0.00663	0.315 *	0.042 *	0.034
	(0.608)	(0.032)	(0.0359)	(0.210)	(0.023)	(1.336)
Employment (binary)	3.409	4.595 ***	0.684	6.704	1.138 *	0.715
	(2.677)	(2.545)	(2.321)	(9.133)	(0.0799)	(0.167)
Constant	136.2 *	114.17 **	77.026 ***	56.93 ***	20.16	13.17
	(342.3)	(28)	(84)	(14.3)	(16.8)	(6.02)
Observations	593	194	149	372	104	54
$\chi^{2}$	6.921 ***	2.022 **	1.348	3.048 ***	1.395	1.876 *
ρ	0.688 **	0.682 ***	0.815 **	0.688 *	0.805 **	0.469 *
σ	2.695	2.657	3.811	2.695	3.682	4.657

Odds ratio and linearized standard errors are reported. Standard errors are reported in parenthesis and clustered at the individual level. High School (HS), General Education Diploma (GED). * *p* < 0.10, ** *p* < 0.05, *** *p* < 0.01; † Shows the base (comparison) category; ^a^ Equals 1 if years are 2010, 2011, 2012, 2013 and 0 if years are 2008 and 2009; ^b^ 1 if years are 2014, 2015 and 0 if years are 2008 and 2009.

**Table 5:** Random effect logit regression of mammography use for all women, by race/ethnicity—MEPS 2008–2015.

	Pre-ACA and Sub-Period A Sample			Pre-ACA and Sub-Period B Sample		
	(Non-Hispanic White)	(Non-Hispanic Black)	(Hispanic)	(Non-Hispanic White)	(Non-Hispanic Black)	Hispanic
Variables	Mammography (1)	Mammography (2)	Mammography (3)	Mammography (4)	Mammography (5)	Mammography (6)
ACA periods (Time dummies)						
Sub period A ^a^	0.822	0.676	1.738	---	---	---
	(0.105)	(0.237)	(0.662)			
Sub Period B ^b^	---	---	---	1.121	2.112	7.20 **
				(0.243)	(1.164)	(4.83)
Breast cancer	1.139	2.223	14.57 **	1.121	2.112	17.20 **
	(0.242)	(1.138)	(15.79)	(0.243)	(1.164)	(19.83)
Period A x breast cancer	2.091 **	2.541 ***	0.107 *	---	---	---
	(0.645)	(0.857)	(0.135)	1.138 *	1.023 **	0.954
Period B x breast cancer	---	---	---	(0.0799)	(0.0114)	(0.0926)
Survivor’s age	1.502 ***	1.562 ***	2.062 ***	1.681 ***	2.448 ***	1.102 ***
	(0.0711)	(0.214)	(0.352)	(0.118)	(0.618)	(0.0302)
Census regions (Northeast †)						
Midwest	0.651 **	0.876	2.135	0.919	0.710	3.251
	(0.134)	(0.502)	(1.468)	(0.274)	(0.700)	(3.705)
South	0.653 **	0.514	0.787	0.897	0.369	0.356
	(0.130)	(0.246)	(0.419)	(0.256)	(0.317)	(0.346)
West	0.532 ***	0.369	1.065	0.586 *	0.356	1.345
	(0.111)	(0.231)	(0.557)	(0.174)	(0.377)	(1.231)
MSA (non-MSA †)	0.984	1.039	3.898 ***	1.046	0.488	17.91 ***
	(0.142)	(0.481)	(1.980)	(0.227)	(0.411)	(16.57)
Marital status (Married †)						
Widow/divorced	0.656 ***	0.735	0.355 **	0.589 ***	1.080	0.231 **
	(0.0850)	(0.283)	(0.144)	(0.113)	(0.610)	(0.157)
Never married	0.587 **	0.872	0.264 ***	0.672	2.076	0.257 *
	(0.141)	(0.399)	(0.132)	(0.269)	(1.488)	(0.208)
Education (HS & GED †)						
Bachelor	1.482 **	1.396	1.908	1.478	0.611	2.622
	(0.235)	(0.623)	(1.157)	(0.376)	(0.494)	(3.403)
Graduate	1.998 ***	1.388	1.914	3.073 ***	0.976	0.951
	(0.382)	(0.743)	(1.624)	(0.978)	(0.848)	(1.322)
Number of priority conditions	1.031	0.967	1.485 **	1.031	1.042	1.550 *
	(0.0431)	(0.0995)	(0.240)	(0.0637)	(0.197)	(0.396)
Family income as % FP line (Low income †)						
Middle income	1.413 **	1.253	0.897	1.231	0.931	0.442
	(0.198)	(0.462)	(0.381)	(0.261)	(0.577)	(0.317)
High income	2.265 ***	1.489	1.598	1.699 **	0.721	4.430
	(0.373)	(0.770)	(1.063)	(0.402)	(0.550)	(5.220)
Employment (Binary)	1.567 ***	1.093	1.188	1.876 ***	3.436 **	0.961
	(0.231)	(0.396)	(0.488)	(0.402)	(2.071)	(0.613)
Constant	0.369 ***	0.649	0.0222 ***	0.152 ***	0.0732	0.00277 ***
	(0.136)	(0.706)	(0.0256)	(0.0821)	(0.130)	(0.00556)
Observations	2402	424	344	1138	186	161
$\chi^{2}$	197.6 ***	35.50 ***	59.26 ***	100.5 ***	23.20 ***	31.19 ***
ρ	9.22 **	2.01 *	2.78 **	2.14 *	3.92 ***	9.01 ***
σ	0.00551	0.00257	0.00302	0.00839	0.00359	0.000544

Odds ratio and linearized standard errors are reported. Standard errors are reported in parenthesis and clustered at the individual level. High School (HS), General Education Diploma (GED). * *p* < 0.10, ** *p* < 0.05, *** *p* < 0.01; † Shows the base (comparison) category; ^a^ Equals 1 if years are 2010, 2011, 2012, 2013 and 0 if years are 2008 and 2009; ^b^ Equals 1 if years are 2014, 2015 and 0 if years are 2008 and 2009.

**Table 6:** Conditional Poisson-log normal hurdle model of physician’s visits among breast cancer survivors—MEPS 2008–2015.

	Pre-ACA and Sub-Period A Sample			Pre-ACA and Sub-Period B Sample		
	(Non-Hispanic White)	(Non-Hispanic Black)	(Hispanic)	(Non-Hispanic White)	(Non-Hispanic Black)	Hispanic
Variables	Physician Visits (1)	Physician Visits (2)	Physician Visits (3)	Physician Visits (4)	Physician Visits (5)	Physician Visits (6)
ACA periods (Time dummies)						
Sub period A ^a^	–0.201 ***	–1.368 ***	–0.403 *	---	---	---
	(0.0739)	(0.455)	(0.228)			
Sub Period B ^b^	---	---	---	−0.111	1.814 **	0.0302
				(0.0815)	(0.769)	(0.237)
Survivor’s age	–0.0568	–0.130 **	–0.200 ***	−0.00735	–0.0658	–0.0941
	(0.0350)	(0.0568)	(0.0739)	(0.0367)	(0.0698)	(0.108)
Census regions (Northeast †)						
Midwest	–0.113	–0.139	–0.238	–0.00516	1.299 ***	0.603
	(0.105)	(0.260)	(0.335)	(0.119)	(0.314)	(0.367)
South	–0.183 *	–0.220	1.354 ***	−0.0503	2.073 ***	0.524
	(0.101)	(0.206)	(0.412)	(0.107)	(0.740)	(0.542)
West	–0.209 *	(0.409)	0.595 **	–0.320 ***	–0.132	0.454 ***
	(0.111)	0.166	(0.303)	(0.104)	(0.237)	(0.148)
Marital status (Married †)						
Widow/divorced	–0.0970	(0.325)	1.613 ***	0.0316	–0.191	0.612 ***
	(0.0801)	–0.372	(0.261)	(0.0885)	(0.281)	(0.178)
Never married	0.0278	0.112	–0.287 **	0.314	–0.280	0.871 ***
	(0.168)	(0.147)	(0.143)	(0.300)	(0.287)	(0.264)
Education (HS & GED †)						
Bachelor	0.0314	0.0905	0.408	0.0227	1.023	–0.326 **
	(0.0954)	(0.185)	(0.381)	(0.107)	(1.170)	(0.140)
Graduate	0.374	–0.250 **	1.023	–0.326 **	–0.0555	–0.186
	(0.662)	(0.108)	(1.170)	(0.140)	(1.867)	(0.147)
Number of priority condition	0.157 ***	0.124 **	0.0401	0.156 ***	0.0715	0.0182
	(0.0257)	(0.0506)	(0.0831)	(0.0279)	(0.0570)	(0.117)
Family income as % FP line (Low income †)						
Middle income	0.0738	–0.0949	–0.424	0.528	0.00200	–0.365
	(0.290)	(0.140)	(0.266)	(0.604)	(0.201)	(0.415)
High income	0.00329	–0.180	–0.481 *	–0.0138	–0.187	–0.250 **
	(0.0948)	(0.165)	(0.263)	(0.112)	(0.206)	(0.108)
Constant	2.243 ***	2.424 ***	3.598 ***	1.774 ***	2.157 ***	0.360
	(0.269)	(0.408)	(0.601)	(0.292)	(0.484)	(1.271)
Observations	653	221	111	480	133	82
Log-Likelihood value	–2035	–665.9	–349.5	–1521	–390.6	–264.1
$\chi^{2}$	56.53 ***	14.06 ***	29.29 ***	42.11 ***	5.330 ***	25.34 ***
Vuong-Test	2.098	3.462	–0.0109	1.939	−0.434	0.633
σ	0.814	0.772	0.831	0.767	0.772	0.879

Marginal effects and linearized standard errors are reported. Standard errors are reported in parenthesis and clustered at the individual level. * *p* < 0.10, ** *p* < 0.05, *** *p* < 0.01; † Shows the base (comparison) category; To be consistent with Figure 3, we only report the second hurdle. The first hurdle (Probit model) is available on request. ^a^ Equals 1 if years are 2010, 2011, 2012, 2013 and 0 if years are 2008 and 2009; ^b^ Equals 1 if years are 2014, 2015 and 0 if years are 2008 and 2009.

**Table 7:** Box-cox regression model of prescription drug expenditures, by race/ethnicity—MEPS 2008–2015.

	Non-Hispanic White	(Non-Hispanic Black)	(Hispanics)
Variables	Prescription Drug Expenditure	Prescription Drug Expenditure	Prescription Drug Expenditure
Time (1 after ACA, 0 before)	–0.949 *	–0.878 **	–0.946 *
	(0.490)	(0.399)	(0.510)
Age (divide by 10)	0.386 **	0.203	–0.733
	(0.195)	(0.322)	(0.550)
Census regions (Northeast †)			
Midwest	0.604 **	–0.679	–2.259
	(0.279)	(1.341)	(1.506)
South	–0.452	–1.099	–0.720
	(0.299)	(1.075)	(0.963)
West	0.0512	–2.928 *	–2.696 ***
	(0.716)	(1.492)	(0.934)
Marital status (Married †)			
Widow/divorced	–0.487	0.560 **	–0.241
	(0.483)	(0.255)	(0.762)
Never married	0.0433	1.016 ***	0.744
	(0.989)	(0.249)	(1.235)
Education (HS & GED †)			
Bachelor	–0.263	0.0785	–0.666
	(0.528)	(0.906)	(0.879)
Graduate	0.0911	–0.600	–4.598 ***
	(0.678)	(1.285)	(1.669)
Npriority	1.985 ***	1.383 ***	1.182 ***
	(0.159)	(0.256)	(0.313)
Family income as % FP line (Low income †)			
Middle income	–0.660	–0.274	–0.688
	(0.583)	(0.907)	(0.824)
High income	–0.0468	1.075	–0.784
	(0.571)	(1.048)	(0.994)
Constant	9.926 ***	10.16 ***	9.423 ***
	(1.483)	(2.237)	(1.777)
Observations	941	303	174
R^2^	0.187	0.124	0.218
RSS	41,584	11,837	3297
MSS	9580	1673	918.8
RMSE	6.694	6.389	4.526
F-Statistics	17.82	3.415	3.739

Standard errors are reported in parenthesis and clustered at individual level. * *p* < 0.10, ** *p* < 0.05, *** *p* < 0.01; † Shows the base (comparison) category.

## Appendix A

**Table A1:** Result of logistic regression analysis of health insurance coverage—MEPS 2008–2015.

Variables	Non-Hispanic White	Non-Hispanic Black	Hispanics
	(Insurance Coverage)	(Insurance Coverage)	(Insurance Coverage)
Time (1 after ACA, 0 otherwise)	0.772	4.855 *	2.720 **
	(0.349)	(3.956)	(1.323)
Survivor’s age	2.016 ***	1.621	2.779 ***
	(0.403)	(0.712)	(1.096)
Census regions (Northeast)			
Midwest	0.243	1.610 ***	0.828
	(0.273)	(0.288)	(1.282)
South	0.127 *	2.720 **	0.247
	(0.137)	(1.32)	(0.278)
West	0.120 *	0.130 ***	0.216 ***
	(0.134)	(0.1032)	(0.074)
Marital status (Married)			
Widowed/divorced	1.150	0.962	1.545
	(0.551)	(1.078)	(1.234)
Never married	0.357	2.968	2.504
	(0.241)	(4.273)	(2.959)
Educational level (GED and HS)			
Bachelor	3.621 **	1.545	0.997
	(2.009)	(0.655)	(0.893)
Graduate	2.187	2.405	0.102 ***
	(1.472)	(1.550)	(0.0605)
Health status (Excellent/very good)			
Good/fair	0.851	0.898	2.550
	(0.379)	(0.901)	(2.214)
Poor	2.192	0.789	0.441
	(2.480)	(1.288)	(0.593)
Number of priority conditions	1.096	1.201	1.417
	(0.192)	(0.434)	(0.558)
Family income as % FPL (low income)			
Middle income	0.525	0.732	0.119 ***
	(0.278)	(0.749)	(0.084)
High income	1.690	0.3152 ***	2.215
	(1.053)	(0.066)	(2.844)
Employment status	0.672	0.596	2.281
	(0.332)	(0.576)	(2.050)
Constant	2.407	0.255	0.0249
	(4.115)	(0.747)	(0.0599)
Observations	926	389	298
LR $\chi^{2}$	48.67 ***	22.60 ***	12.18 ***

Odds ratio reported for all the coefficients. Balanced repeated replication is applied to take into consideration of complex survey design. Standard errors are reported in parenthesis and clustered at individual level. High School (HS), General Education Diploma (GED); * *p* < 0.10, ** *p* < 0.05, *** *p* < 0.01.

## Appendix B

**Table A2:** Result of Logistic regression of Mammography use among racial and ethnic groups—MEPS 2008–2015.

Variables	Non-Hispanic White	Non-Hispanic Black	Hispanics
	(Mammography)	(Mammography)	(Mammography)
Time (1 after ACA, 0 otherwise)	1.909 **	1.307 **	0.318 **
	(0.553)	(0.588)	(0.160)
Age of survivors	1.038	1.006	1.470
	(0.149)	(0.280)	(0.780)
Census regions (Northeast)			
Midwest	0.390 *	0.387	0.181
	(0.199)	(0.500)	(0.709)
South	0.584	0.120 ***	3.883 **
	(0.293)	(0.0834)	(1.95)
West	0.312 **	1.501 *	1.897
	(0.160)	(0.337)	(5.051)
MSA (non-MSA)	0.933	0.643	0.308
	(0.335)	(0.724)	(0.495)
Marital status (Married)			
Widowed/divorced	0.813	0.243	4.989
	(0.258)	(0.216)	(7.779)
Never married	0.399	0.828	0.425
	(0.246)	(1.084)	(0.553)
Educational level (GED and HS)			
Bachelor	0.473 **	0.918	1.475
	(0.160)	(0.396)	(2.538)
Graduate	3.023 *	2.113 *	0.628
	(1.911)	(0.904)	(1.009)
Number of priority conditions	0.942	1.372	0.329 *
	(0.0942)	(0.344)	(0.196)
Family income as % FPL (low income)			
Middle income	0.882	1.053	0.0342 **
	(0.320)	(0.842)	(0.0142)
High income	0.911	0.476	2.324
	(0.342)	(0.426)	(1.317)
Employment status	2.056 *	1.282 *	0.794
	(0.817)	(0.684)	(1.227)
Constant	13.20 **	47.28	199.1
	(15.55)	(117.3)	(795.4)
Observations	593	194	79
LR $\chi^{2}$	15.66 ***	11.12 **	10.06 **

Odds ratio reported for all the coefficients. Balanced repeated replication is applied to take into consideration of complex survey design. Standard errors are reported in parenthesis and clustered at individual level. High School (HS), General Education Diploma (GED); * *p* < 0.10, ** *p* < 0.05, *** *p* < 0.01.

## Appendix C

**Table A3:** Result of conditional regression analysis model of Physician’s Visits among Breast Cancer Survivors—MEPS 2008–2015.

Variables	(Office-Based Physician Visits)	(Office-Based Physician Visits)	(Office-Based Physician Visits)
	Non-Hispanic White	Non-Hispanic Black	Hispanic
ACA (Time dummy)	−1.329 *	0.987	−1.910
	(0.689)	(1.302)	(2.500)
Survivor’s age	−0.102	−1.097 *	−1.109
	(0.292)	(0.581)	(0.833)
Census regions (Northeast)			
Midwest	−0.0366	−0.917	−4.556 **
	(0.825)	(2.191)	(1.980)
South	−0.138	−1.528	1.765
	(0.798)	(1.936)	(2.094)
West	−0.715	−0.286	1.493
	(0.807)	(2.301)	(2.400)
Marital status (Married)			
Widowed/divorced	−6.452 ***	0.248	−0.757
	(2.348)	(1.106)	(1.775)
Never married	−3.551 **	2.614	2.409
	(0.780)	(2.312)	(3.091)
Educational level (GED and HS)			
Bachelor	2.173 ***	2.455 ***	0.852
	(0.790)	(0.621)	(2.188)
Graduate	2.370 ***	3.767 ***	−0.820
	(0.882)	(1.351)	(2.821)
Number of priority conditions	0.998 ***	0.461	−0.164
Family income as % FPL(low income)	(0.218)	(0.384)	(1.048)
Middle income	−0.803	−0.394	−0.544
	(0.852)	(1.559)	(1.810)
High income	−0.715	−0.367	4.401
	(0.823)	(1.513)	(2.767)
Constant	7.452 ***	11.71 ***	12.10 **
	(2.241)	(3.742)	(5.534)
Observations	857	271	153
R-squared	0.075	0.111	0.130
RSS	62,960	20,034	14,865
MSS	5108	2513	2223
RMSS	8.657	8.881	10.45
F-statistics	13.904 ***	11.426 ***	8.331 **
Number of clusters	857	271	153

Standard errors are reported in parenthesis and clustered at individual level. High School (HS), General Education Diploma (GED). * *p* < 0.10, ** *p* < 0.05, *** *p* < 0.01.

## Appendix D

**Table A4:** Result of Regression Analysis for out-of-pocket prescription drug expenditures—MEPS 2008–2015.

Variables	Non-Hispanic White	(Non-Hispanic Black)	(Hispanics)
	Prescription Drug Expenditure	Prescription Drug Expenditure	Prescription Drug Expenditure
Time (1 after ACA, 0 before)	73.55	−1784 *	−705.3
	(341.6)	(1025)	(1409)
Survivor age	−358.1	−72.83	−1079
	(253.7)	(157.0)	(891.9)
Census regions (Northeeast †)			
Midwest	−112.8	−1784 *	−705.3
	(514.5)	(1025)	(1409)
South	164.0	−1351	−2042
	(568.8)	(1122)	(1242)
West	−432.6	−900.0	4474
	(445.7)	(1026)	(5323)
Marital status (Married †)			
Widow/divorced	51.57	−1886	−740.7
	(386.5)	(1232)	(1710)
Never married	−1095	−3064	995
	(1097)	(2727)	(1097)
Education (HS & GED †)			
Bachelor	−62.62	−647.6	4566
	(376.1)	(665.0)	(4985)
Graduate	−57.57	−868.9	−4542
	(491.0)	(1030)	(2914)
Number of priority conditions	1084 ***	538.2 **	1291 **
	(270.0)	(210.6)	(541.8)
Family income as % FP line (Low income †)			
Middle income	561.2	−1319	−6349
	(461.7)	(907.0)	(7642)
High income	304.4	−1176	−6881
	(381.7)	(777.0)	(8409)
Constant	2951 **	4959 **	9988
	(1365)	(2416)	(6932)
Observations	941	303	174
R-squared	0.074	0.077	0.057
RSS	2.900 × 10^10^	4.940 × 10^9^	4.370 × 10^10^
MSS	2.300 × 10^9^	4.140 × 10^8^	2.640 × 10^9^
RMSE	5591	4126	16,471
F-statistics	5.233 **	1.534	1.155
Number of clusters	941	303	174

Standard errors are reported in parenthesis and clustered at individual level. High School (HS), General Education Diploma (GED). * *p* < 0.10, ** *p* < 0.05, *** *p* < 0.01; † Shows the base (comparison) category.
